# Retinal specific measurement of dark-adapted visual function: validation of a modified microperimeter

**DOI:** 10.1186/1471-2415-11-5

**Published:** 2011-02-08

**Authors:** Michael D Crossland, Vy A Luong, Gary S Rubin, Fred W Fitzke

**Affiliations:** 1 UCL Institute of Ophthalmology, London, UK; 2 NIHR BMRC for Ophthalmology, London, UK

## Abstract

**Background:**

Scotopic function is an important marker of many retinal diseases and is increasingly used as an outcome measure in clinical trials, such as those investigating gene therapy for Lebers congenital amaurosis. Scotopic visual function has traditionally been measured using an adapted perimetry system such as the Humphrey field analyser (HFA). However this system does not control for fixation errors or poor fixation stability. Here we evaluate the use of an adapted microperimeter to measure visual function at defined retinal regions under scotopic conditions.

**Methods:**

A MP-1 microperimeter (Nidek Technologies, Italy) was modified by adding a 1 log unit Neutral Density filter and a 530nm shortpass filter within the optical path of the instrument. Stray light was shielded. Fine matrix mapping perimetry was performed on five younger (<35 years) and five older (>65 years) subjects with no eye disease and good vision. All subjects were fully dark adapted before testing and pupils were dilated with 1% tropicamide. Tests was performed once on the modified MP-1 microperimeter and once using a modified HFA, in a counterbalanced order.

**Results:**

A foveal scotopic scotoma with a sensitivity reduction of >1 log unit was found using each instrument. In addition, the MP-1 system showed the retinal location of the foveal scotoma. Mean test time was 25 minutes for the MP-1 and 32 minutes for the HFA.

**Discussion:**

A modified MP-1 microperimeter can be used to measure scotopic retinal function, creating results which are comparable to the modified Humphrey field analyser. Advantages of the MP-1 system include the ability to track the retina through testing, retinal localisation of the scotoma and a faster test time.

## Background

Rod photoreceptor function is reduced in many retinal diseases including retinitis pigmentosa [1-3], rod-cone dystrophy [4], retinal telangiectasia [5] and congenital stationary night blindness [6]. Recent histological evidence shows that rods may be affected prior to cone photoreceptors in the early stages of age-related macular disease (AMD)[7] and psychophysical data also show a selective impairment of parafoveal rod photoreceptors in AMD [8]. Consistent with this, people with AMD report particular difficulty with vision under dark-adapted conditions [9].

Currently, rod function is measured in clinical studies by measuring the scotopic electroretinogram [10,11], by measuring dark adaptation [12,13], or by performing dark-adapted perimetry [14]. The instrument most currently used for performing dark-adapted perimetry is a modified first-generation Humphrey Field Analyser (HFA, Carl Zeiss Meditec Inc, USA) [8,14-18]. This instrument relies on technology first developed in the 1980 s [19] such as 5.25" floppy discs and a 'light-pen' to enter data. More significantly, it does not correct for poor or unstable fixation. Although the second generation HFA does include an infrared eyetracker and is still commercially available, a custom chipset is required to over ride the self-calibration programme [20]. Further, this instrument does not measure the position of gaze, only changes in gaze from the initial calibration position. The instrument can, therefore, identify poor fixation but can not correct for it.

In one conference abstract it has been reported that scotopic perimetry has been performed with a confocal Scanning Laser Ophthalmoscope [21] but to the best of our knowledge this has not been repeated by others.

The MP-1 microperimeter (Nidek Technologies, Italy), launched in 2002, is a system which performs retinal-specific microperimetry [22,23]. It comprises an infrared camera which provides a retinal image, updated at 25 Hz, and a LCD display which can be used to present stimuli. It also incorporates a fundus camera to capture a full-colour retinal image, and inbuilt software can superimpose the microperimetry plot onto the retinal image. This software includes several perimetry strategies (including the Humphrey 10-2 strategy), and it is straightforward to program new paradigms by specifying the test locations, target size, target exposure duration, background luminance, thresholding strategy and fixation target.

Under fully dark-adapted conditions, a central scotopic scotoma exists in people with healthy retinas, corresponding to the foveal rod-free region. This rod-free region measures about 0.35 mm and subtends approximately 1.25° [24]. Peak rod density is reached at approximately 5° eccentricity, so a relative dark-adapted scotoma can be found within the central 10° of retina.

Here we discuss modifications which can be made to the Nidek MP-1 microperimeter to enable scotopic microperimetry to be performed with this instrument. We show that the technique is operating under scotopic conditions by measuring the foveal scotopic scotoma in ten subjects. We also compare these data to those collected with the modified HFA.

## Methods

### Modification of the instrument

**Figure 1:**
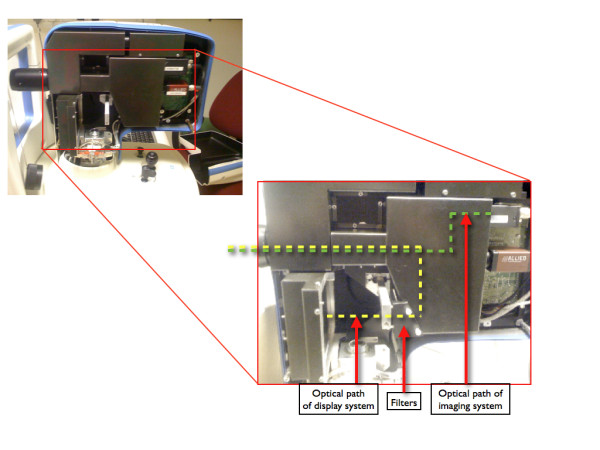
**The optical path and location of filters in the modified MP-1 microperimeter**.

One MP-1 microperimeter was modified for the purpose of this experiment. First, screen luminance was reduced by inserting a 2.0 log unit neutral density filter (NT48-097, Edmund Optics, Barrington, NJ) in the optical path of the LCD monitor on the instrument. Next, stimuli were limited to blue by placing a 500 nm shortpass filter (NT30-635, Edmund Optics, Barrington, NJ) in the same position. The design of the MP-1 enables placement of filters in the optical path of the stimulus display without affecting the imaging system (Figure 1).

Stray light was shielded from the observer by placing black silk covers over vents on the MP-1 which were found to leak light, by removing all LEDs from the MP-1 and its control computer (other than the main power switch which was covered with opaque black tape) and by draping felt sheets around the chin and forehead rest of the device.

The instrument was used in a darkroom laboratory where the ambient light was measured to be <0.1 lux.

### Evaluation

Five younger (under 35 years of age) and five older (over 60 years) subjects participated in the study. Subjects were recruited from family and friends of the authors. No subjects had any history of eye disease and all had visual acuity of 6/6 (20/20, 0.0 logMAR) or better (with spectacle correction if required).

One eye of each subject was dilated using 1.0% tropicamide. Dark adaptation was performed in a dark room (<0.1 lux) with an opaque eyepatch over the eye to be tested for at least thirty minutes. Subjects were encouraged to listen to a radio station of their choice during dark adaptation.

Perimetry was performed in counterbalanced order on the MP-1 and HFA. The untested eye was occluded in all cases.

On the MP-1 microperimeter, fine matrix mapping was performed using 100 points arranged regularly within a square of side length 10° centred at the fixation centre. Stimuli were Goldmann size III targets (4 mm^2^) [25], presented for 250 msec. Sensitivity at each point was determined using a 4-2 strategy. This means that if a point is seen at a given instensity, the next stimulus presentation at that location was 4dB fainter until it is not seen, following which intensity increases by 2dB until it is seen again [25]. The fixation target used was a circle of 15° diameter. The fixation target was this large to avoid it being superimposed onto the stimulus grid, potentially affecting stimulus detection.

The HFA was modified in the manner described by Jacobson *et al *[14]. The large diamond LED fixation target was used. Stimulus properties were identical to those on the MP-1 microperimeter.

After all data collection was completed, the room lighting was increased and a colour photo was taken on the MP-1 microperimeter for superimposing the microperimetry plot.

Scotoma depth was defined as being the difference between the highest and lowest threshold for each test.

The study was approved by the UCL ethics committee and conformed to the Declaration of Helsinki. All subjects gave their informed consent prior to data collection.

## Results

**Figure 2:**
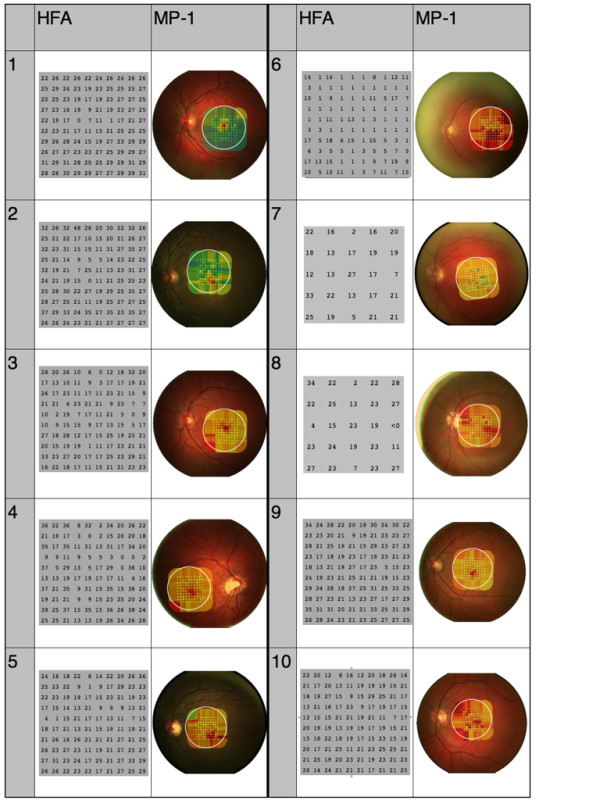
**Results from both instruments for each subject**. HFA plots: numbers show threshold sensitivity, in decibels attenuation, at each point within the visual field (larger numbers show better function). MP-1 plots: colours show threshold sensitivity (bright green shows best function, deep red shows poorest function).

A central scotopic scotoma of at least 1 log unit was identified in all subjects on both tests. Figure 2 shows all of the HFA and MP-1 plots. For subjects 7 and 8 instrument failure or subject fatigue lead to the Humphrey test being abandoned after the first set of data were collected (25 points). These data were excluded from the analysis of test time.

Mean scotoma depth was 31dB on the HFA and 13dB on the MP-1 method (matched pairs, p < 0.0001). There was a correlation between the depth of the scotoma on the HFA and MP-1 (Spearman Rho = 0.51).

The MP-1 indicated that this scotoma was coincident with the foveal centre in all subjects.

However, the point of minimum sensitivity was frequently distant from the centre of the HFA visual field plot, which we assume to be due to the lack of fixation control on this instrument.

The MP-1 test was quicker than the HFA: mean test time was 1914 seconds (31 minutes 55 seconds) for the HFA and 1526 seconds (25'26") for the MP-1. This difference was statistically significant (matched pairs, p < 0.01).

## Discussion

We have demonstrated that the MP-1 microperimeter can be adapted to perform retinal-specific scotopic perimetry in a population of younger and older adults with no eye disease. The adaptation to our MP-1 was straightforward and cost approximately £300 ($450, €335).

The depth of the scotoma measured using the MP-1 is significantly less than that measured using the HFA. We assume that this is due to the low dynamic range of the LCD display in the MP-1 microperimeter. This display is only able to show stimuli across a 2 log unit range of luminance, meaning that the largest scotoma depth which can be measured is 20dB. Some subjects (for example subjects 1, 2, 5 and 7) were able to see the dimmest stimuli which can be presented under this condition (20dB attenuation, shown as a filled green square in the MP-1 image), meaning that dimmer stimuli are required in order to accurately measure threshold sensitivity. In contrast, the modified Humphrey is able to present stimuli over a greater range and the depth of scotoma measured was up to 48dB (for subject 2). As screen technology increases it is likely that a display which allows a greater dynamic range can feasibly be used in this instrument. Alternatively, a system which can automatically add or remove filters would extend the dynamic range of the microperimeter.

A further limitation of the reduced dynamic range of the MP-1 microperimeter is that the range of luminance with the filters we have used may not be sufficient to measure the scotopic visual function of people with eye disease: a floor effect may be apparent where none of the targets are visible. After several control experiments we found a 2 log unit neutral density filter to be the most suitable to measure scotopic thresholds in our control subjects. We plan to perform scotopic perimetry on subjects with eye disease in the future to determine the optimal filter system for this patient group. It may be that a neutral density filter which transmits more light is needed for this population. Before this instrument can be used as a clinical tool, population norms will need to be defined for people with and without eye disease. Test-retest variability in threshold quantification and scotoma parameters is also not yet known, and will be investigated in future studies. The correlation between scotopic microperimetry and imaging or electrophysiology tests also requires investigation.

The image produced on the MP-1 microperimeter shows that the scotopic scotoma is coincident with the foveal centre in all cases. For some subjects (for example, subject 2) the stimulus grid is not centred on the fovea. This is because the grid position is centred assuming that the point of fixation is coincident with the foveal centre. It is known that the "scotopic fovea" used for fixation under dark-adapted conditions is variable between subjects but tends to be in superior retina [26]. This would have the effect of decentring the grid upwards, as can be seen in Figure 2. A strength of the MP-1 technique is that this decentration is corrected for once the sensitivity map is superimposed on the colour image.

It is significant that the region of minimum sensitivity is often far from the centre of the HFA visual field plot, meaning that it is impossible to relate the visual field plot to retinal features. We used the "large diamond" fixation target for this test, which is known to induce poorer fixation stability than other targets [27]. To investigate whether fixation is poorer under dark-adapted conditions, we measured fixation stability using the MP-1 for the younger subjects whilst performing the same test under photopic conditions. Mean fixation stability was 22 600 minarc^2 ^under dark adapted conditions and 6880 minarc^2 ^for the same test under photopic conditions (matched pairs, p < 0.05). The fixation target used was very large, and we asked subjects to look towards the centre of the circle rather than to use it as a fixation guide. As the MP-1 tracks eye movement during microperimetry errors and offsets in fixation would be corrected for. However, it would be of concern if eye movements were made during stimulus presentation. Further, our fixation target is blue due to the filter system used in the instrument modification. Whilst a red target would be optimal, this is not possible using our instrument modification.

The MP-1 test was significantly quicker than the HFA technique. As our tests were performed in counterbalanced order, this difference in time is unlikely to be due to subject fatigue. Rather, it is likely to be due to the 'pre-test' function of the MP-1 which analyses sensitivity in four locations and selects the initial stimulus intensity in each quadrant of retina on the basis of this value. Although the newer Humphrey field analyser also performs this test, it is not performed on our first generation HFA. The test is still very lengthy. This is largely as we used a dense grid of 100 points to match our stimulus set to the 'fine matrix mapping' technique of Fitzke and others [16]. It is possible to reduce the test time further by testing fewer points (for example, to investigate function over a retinal lesion only). This is more straightforward on the MP-1 than the HFA.

## Conclusions

The MP-1 microperimeter can be adapted to measure dark-adapted visual function.

The 'depth' of the scotoma measured using the MP-1 is less than when measured using the HFA: we assume this is due to the lower dynamic range of the MP-1's LCD display.

The location of the scotopic scotoma is offset on the HFA technique, probably secondary to reduced fixation stability under dark-adapted conditions.

The MP-1 method is quicker than the modified HFA technique; it corrects for poor fixation; and it shows the retinal position of the scotopic scotoma.

## Competing interests

The authors declare that they have no competing interests.

## Authors' contributions

MDC performed the modification of the instrument, collected the data, performed the statistical analyses and drafted the manuscript. VAL assisted in the data collection and in the programming of the HFA. GSR and FWF participated in study design and critically appraised the manuscript. All authors read and approved the final manuscript.

## Pre-publication history

The pre-publication history for this paper can be accessed here:

http://www.biomedcentral.com/1471-2415/11/5/prepub
